# Reproducibility and repeatability of same-day two sequential FDG PET/MR and PET/CT

**DOI:** 10.1186/s40644-017-0113-9

**Published:** 2017-04-05

**Authors:** David Groshar, Hanna Bernstine, Natalia Goldberg, Meital Nidam, Dan Stein, Ifat Abadi-Korek, Liran Domachevsky

**Affiliations:** 1 Department of Nuclear Medicine, Assuta Medical Center, 20 habarzel st., 6971028 Tel-Aviv, Israel; 2 Sackler Faculty of Medicine, Tel Aviv University, Tel-Aviv, Israel

**Keywords:** PET/MR, SUV, Reproducibility, Repeatability, Reliability

## Abstract

**Background:**

To determine PET/CT and PET/MR reproducibility and PET/MR repeatability of fluorine 18 fluorodeoxyglucose (FDG) uptake measurements in tumors in cancer patients.

**Methods:**

This IRB approved prospective study was performed between October 2015 and February 2016 in consecutive patients who performed same day PET/CT and two sequential PET/MR. Thirty three patients with visible tumors (*N* = 63) were included. SUV for body weight (SUV) and lean body mass (SUL) were obtained. Volume of interest (VOI) with a threshold of 40% was used and SUV/L’s, metabolic tumor volume (MTV) and tumor to liver ratio (T/L) were calculated. Measurements were plotted in a scattered diagram to visually identify correlation, a regression line was drawn and the equation of the line was calculated. Bland-Altman plots expressed as percentages were constructed to assess the agreement between measurements. The maximal clinically acceptable limits range was defined as ±30%.

**Results:**

Lesional SUV’s, SUL’s and MTV corrected to body weight (BW) and lean body mass (LBM) demonstrated strong positive linear correlation between PET/CT and PET/MR and between two sequential PET/MR. The 95% limits of agreement ranged from -27.7 to 17.5 with a mean of -5.1 and -27.6 to 17.9 with a mean of -4.9 for SUVpeak and SULpeak, respectively for sequential PET/MR. Other PET metrics demonstrated limits range that is above ±30% between PET/CT and PET/MR and between two sequential PET/MR.

**Conclusion:**

PET/MR SUV/L peak has a clinically acceptable repeatability performance and can be used to evaluate the response to treatment.

## Background

The introduction of hybrid PET/MR imaging offers a new modality that combines high soft-tissue contrast resolution of MR with metabolic imaging from PET within a single imaging session. This modality has shown promising results in oncological imaging and could be useful in the management of patients with cancer [1]. Quantitative or semi-quantitative imaging biomarkers such as fluorine 18 fluorodeoxyglucose (FDG) may predict response to therapy earlier compared to conventional imaging as metabolic changes in tumors may precede changes in tumor size and texture and determine tissue viability [2].

FDG uptake can be assessed qualitatively as mild, moderate or intense compared to the background uptake in normal appearing tissues of which liver parenchyma is the most commonly used. However, quantitative or semi-quantitative PET metrics, rather than qualitative assessments, should be used in order to obtain comparable results both from sequential studies of a single patient and between different patient groups [3]. Indeed, SUV that is a semi quantitative measurement to evaluate FDG uptake in a tumor or organ by PET/CT has been successfully used in clinical studies in addition to visual assessments.

The use of semi-quantitative measurements for patient follow-up or for comparison between different scanners relies on the high degree of repeatability and reproducibility, respectively. Knowledge of the expected range in reproducibility and repeatability is needed to determine what change in parameters between two examinations can be considered significant in an individual patient or between patient groups. Commercially available Dixon-based PET/MR attenuation correction (MRAC) differs from density-based PET/CT attenuation correction (CTAC) and has been shown to affect FDG uptake measurements in tumor lesions and in normal appearing structures [4]. Several studies have compared FDG PET images from PET/CT and PET/MR in clinical data [1, 5–13] and found similar diagnostic performance and detection rates, despite some differences in the semi-quantitative assessment of FDG uptake [14]. Unlike previous reports the test-retest repeatability in this study was performed on the same day and patients were randomized regarding the order of PET/CT and PET/MR studies. Same-day repeatability with studies performed in sequence enables evaluation of the PET/MR system reliability as variables related to the patient such as patient habitus or changes in tissues following therapy are similar, while randomization of patients obviates differences in biodistribution which still affect FDG uptake even with a modest temporal offset. The purpose of this observational prospective study is to determine PET/CT and PET/MR reproducibility and test-retest PET/MR repeatability of lesional FDG PET metrics obtained by PET/CT and by two sequential PET/MR examinations performed on the same day in patients with cancer.

## Methods

This observational prospective study was approved by the institutional review board. Informed written consent was obtained from all patients participating in the study. Between October 2015 and February 2016, consecutive patients who performed PET/CT and two sequential non-enhanced whole-body PET/MR were enrolled. All patients had a biopsy-proven cancer (Table 1) and underwent PET/CT either for initial evaluation or for follow-up. Patients were randomized using a simple randomization to a group in which sequential PET/MR was performed first and to a second group in which PET/CT was performed first (Table 1). The sequential PET/MR studies were conducted in a row (i.e., immediately after the first PET/MR scan was ended the second PET/MR was started). Only patients with visible tumor based on PET/CT and PET/MR findings were included. A total of 33 out of 67 patients with 63 conspicuous tumor lesions (mean age 53.1 ± 12.1years, 19 females, mean age 52.4 ± 11.8 years and 14 males, mean age 54.1 ± 12.5 years) were included (Table 1).

**Table 1:** Patient characteristic

		Patients with visible lesions (*N* = 33)
Age (years)		53.1 ± 12.1 (28–75)
Gender	Female	n = 19, age 52.4 ± 11.8
	Male	n = 14, age 54.1 ± 12.5
MR first		16
CT first		17
Time to CT (minutes)		110 ± 32 (47–185)
Time to MR (minutes)		104 ± 36 (41–175)
Time to exam (minutes)		81 ± 22 (41–175)
Time between exams (minutes)		53 ± 17 (25–88)
Blood glucose levels (mg/dl)		95.47 ± 26.4 (69–203)
BMI		25.8 ± 5.1 (16.2–35.7)
Disease	Breast	12
	Lung	7
	Lymphoma	2
	Melanoma	1
	CRC	7
	Head and neck	3
	other	1
Number of lesions		
	1	13
	2	10
	3	10

### PET/CT Protocol

PET/CT was performed using an integrated PET/CT scanner (GEMINI TF, PHILIPS Medical Systems, Cleveland, Ohio, USA). Intravenously FDG dose of 5.18MBq/kg (varied from 370 to 666 MBq) and 800–1000 mL of diluted iodinated contrast material was administered orally for bowel opacification. Contrast-enhanced 64-slice multi-detector CT was performed from skull base to mid-thigh with the arm-up position with tube voltage of 120 kVp, spiral CT at 0.8s per rotation with modulated 30–250 mAs, section thickness of 3.00 mm, and 3.00 mm interval with image reconstruction every 3.0 mm. Intravenous iodine contrast media (Omnipaque 300; iohexol 0.623 g/ml, GE Healthcare, USA; 1.5 cm3/kg) was administered in all examinations, except for patients with known iodine hypersensitivity or renal insufficiency. PET emission images were obtained with 2 min of acquisition per bed position with five to six bed positions from skull base to mid-thigh. PET data was reconstructed using 3D- ordered subset expectation maximization (OSEM), (3 iteration and 20 subsets, 4 mm Gaussian filter) on 144 matrix with CT-based attenuation correction.

### PET/MR Protocol

FDG PET/MR was performed from skull base to mid-thigh with the arm-down position, on the Biograph mMR (Siemens AG, healthcare sector, Erlangen, Germany) simultaneous PET/MR system. Patients were positioned supine and multi-step/multi-bed scanning was performed in caudo-cranial direction with four bed positions. We used a 24 –channel spine RF coil integrated within the MR bed and 3 surface body coils (6 channel each) to cover the thorax, abdomen and pelvis. For the neck we used a 16-channel RF head/neck coil.

PET data was acquired in the list mode and reconstructed with 3D-OSEM, (3 iteration and 21 subsets, 4 mm Gaussian filter) on 172 matrix. Each bed position was started with coronal Dixon-based sequences for MR attenuation correction (MRAC) (breath holding) (19s). This was followed by axial T2 half-fourier acquisition single shot turbo spin echo (HASTE) (free breathing) (36s), coronal T2 HASTE with fat suppression (FS) (Inversion recovery (IR) –based) (44s) and axial T1 volumetric interpolated breath-hold examination (VIBE) Dixon (breath holding) (20s). PET data was acquired simultaneously with acquisition time of 5 min for each bed position. Similar parameters were used for the sequential PET/MR scan.

### Image analysis

We used dedicated software (Syngo.via; Siemens AG, healthcare sector, Erlangen, Germany) for maximal, peak and mean SUV calculations normalized for body weight (SUV) and lean body mass (SUL).

SUV/Lmax is a single-pixel value of the maximal SUV/L within the sphere, whereas SUV/Lpeak is the mean SUV/L within a predetermined volume of interest (VOI) of 1ml around the voxel with the highest SUV/L in the sphere. SUV/Lmean is the average SUV/L value within the sphere.

Normalization for BW was performed using the patient weight in kg, measured before FDG injection and for LBM using the following formula:

$$ \mathrm{L}\mathrm{B}\mathrm{M}\ \left(\mathrm{female}\right) = \left(1.07\ \mathrm{X}\ \mathrm{B}\mathrm{W}\right)\ \left(\mathrm{kg}\right)\ \hbox{--}\ 148\ {\left[\mathrm{BW}\ \left(\mathrm{kg}\right)/\mathrm{body}\ \mathrm{height}\ \left(\mathrm{cm}\right)\right]}^2 $$

$$ \mathrm{L}\mathrm{B}\mathrm{M}\ \left(\mathrm{male}\right) = \left(1.1\ \mathrm{X}\ \mathrm{B}\mathrm{W}\right)\ \left(\mathrm{kg}\right)\ \hbox{--}\ 128\ {\left[\mathrm{BW}\ \left(\mathrm{kg}\right)/\mathrm{body}\ \mathrm{height}\ \left(\mathrm{cm}\right)\right]}^2 $$

Studies were searched for the presence of lesions by visual analysis. Characterization of lesions was performed based on increased FDG uptake compared to surrounding tissue and abnormal structure on CT and MR and was conducted by a dual board-certified in radiology and nuclear medicine physician (L.D., with 3 years of experience) and a board-certified nuclear medicine physician (H.B., with 9 years of PET/CT experience). However, measurements were only conducted by a board-certified nuclear medicine physician (H.B.). There was no lower or upper size limit for any visible lesion.

A spherical VOI was placed in the lesion and an isocontour VOI with a threshold of 40% of SUV/Lmax corrected to LBM and BW was drawn in up to 3 distinct separated lesions (i.e., the largest lesions were selected) per each patient. In addition, a VOI with a diameter of 3 cm was drawn on the right lobe of the liver and tumor to liver ratio was determined. All VOI’s were visually evaluated on axial, sagittal and coronal planes to be certain that the VOI is well located in the desired area.

### Statistical analysis

Values are shown as mean ± SD from sequential PET/MR variables and from variables values from PET/MR and PET/CT. For PET/MR and PET/CT comparison, the average of the two PET/MR measurements was used. Measurements were plotted in a scattered diagram to visually identify correlation, a regression line was drawn and the equation of the line was calculated.

Bland-Altman plots were constructed for each PET metrics variable to assess the agreement between the measurements. The maximal clinically acceptable limits range was defined as ±30%, based on the PERCIST definition for partial response and progressive disease [15].

Statistical analysis was performed using SPSS (IBM version 21) and MedCalc (version 16.2.0).

## Results

### Lesional correlation and agreement between PET/CT and PET/MR

**Fig. 1:**
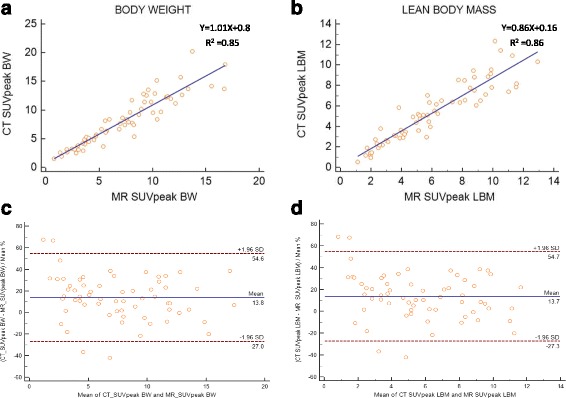
Scatter diagram with regression line and Bland-Altman plots between PET/CT and PET/MR SUVpeak measurements corrected to body weight (**a**, **c**) and lean body mass (**b**, **d**)

A mixed effects model that accounted for correlation of several lesional measurements within a patient showed no significant effect on the results. PET/CT and PET/MR SUV and SUL measurements of lesions, liver and tumor to liver ratio are shown in Table 2. Lesional SUV’s and SUL’s corrected to BW and LBM demonstrated strong positive linear correlation between PET/CT and PET/MR (Fig. 1).

**Table 2:** PET/CT and PET/MR SUV and SUL measurements of tumor, liver and tumor to liver ratio (ratio)

	Tumor	Liver	Ratio
SUV max			
PET/CT	10.54 ± 5.41	2.74 ± 0.57	4.97 ± 2.91
PET/MR	10.02 ± 5.23	2.30 ± 0.69	4.76 ± 3.18
SUV mean			
PET/CT	6.11 ± 3.13	2.17 ± 0.41	3.01 ± 1.81
PET/MR	5.66 ± 2.84	1.80 ± 0.36	3.24 ± 1.69
SUV peak			
PET/CT	8.14 ± 4.32	2.66 ± 0.41	3.21 ± 1.92
PET/MR	7.25 ± 3.95	2.01 ± 0.39	3.75 ± 2.21
SUL max			
PET/CT	7.81 ± 3.97	2.28 ± 0.31	3.58 ± 2.04
PET/MR	7.42 ± 3.85	1.64 ± 0.29	4.74 ± 2.78
SUL mean			
PET/CT	4.5 ± 2.27	1.56 ± 0.25	3.08 ± 1.84
PET/MR	4.17 ± 2.09	1.30 ± 0.24	3.35 ± 1.85
SUL peak			
PET/CT	6.01 ± 3.1	1.92 ± 0.25	3.28 ± 1.94
PET/MR	5.35 ± 2.88	1.46 ± 0.26	3.79 ± 2.23

The 95% limits of agreement and mean difference expressed as percentages for lesional SUV max, peak and mean corrected to BW and LBM were above the clinically acceptable range (Table 4).

**Fig. 2:**
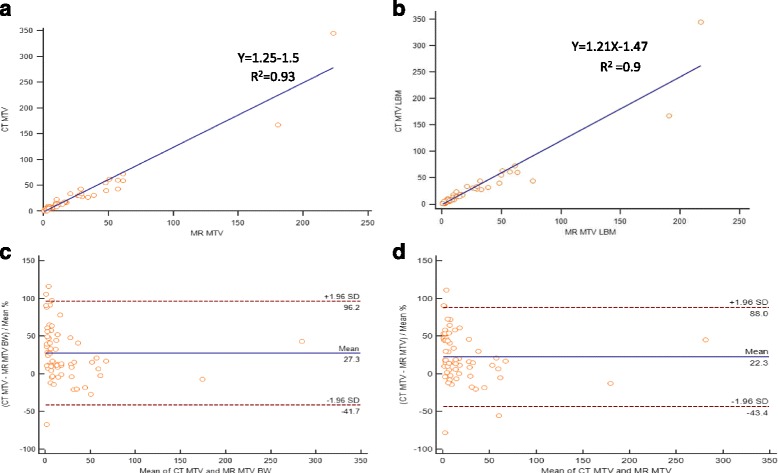
Scatter diagram with regression line and Bland-Altman plot between PET/CT and PET/MR MTV measurements corrected to BW (**a**, **c**) and LBM (**b**, **d**)

Representative Bland-Altman plots for SUVpeak with y-axis values expressed as percentages showed 95% limits of agreement ranging from -27 to 54 with a mean of 13.8 and -27.3 to 54.7 with a mean of 13.7 corrected to BW and LBM, respectively (Fig. 1). Lesional MTV corrected to BW and LBM demonstrated strong linear correlation between PET/CT and PET/MR (Fig. 2). Bland-Altman plots for MTV with y-axis values expressed as percentages showed 95% limits of agreement ranging from -41.7 to 96.2 with a mean of 27.3 and -43.4 to 88 with a mean of 22.3 corrected to BW and LBM, respectively (Fig. 2).

### Lesional correlation and agreement between two sequential PET/MR

**Fig. 3:**
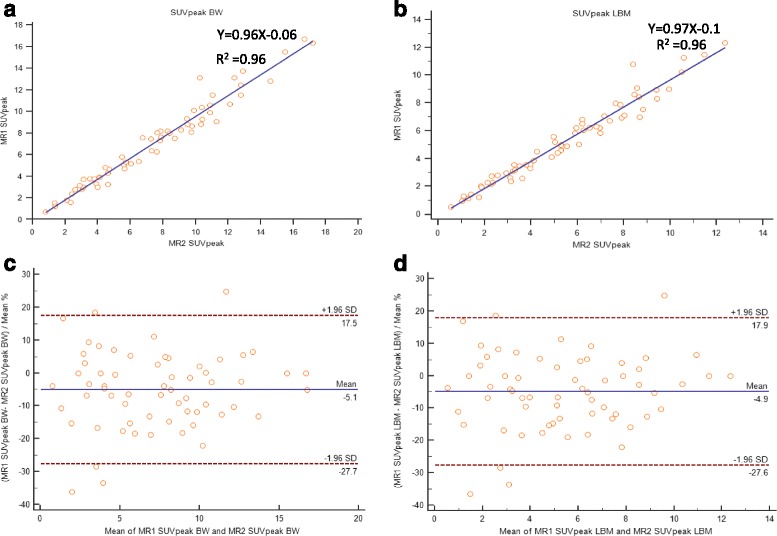
Scatter diagram with regression line and Bland-Altman plots between sequential PET/MR SUVpeak measurements corrected to body weight (**a**, **c**) and lean body mass (**b**, **d**)

**Fig. 4:**
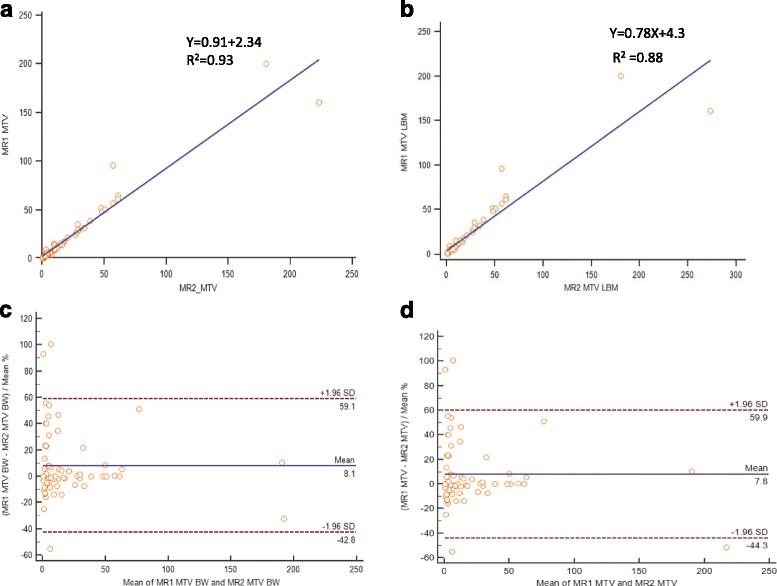
Scatter diagram with regression line and Bland-Altman plot between sequential PET/MR MTV measurements corrected to BW (**a**, **c**) and LBM (**b**, **d**)

**Fig. 5:**
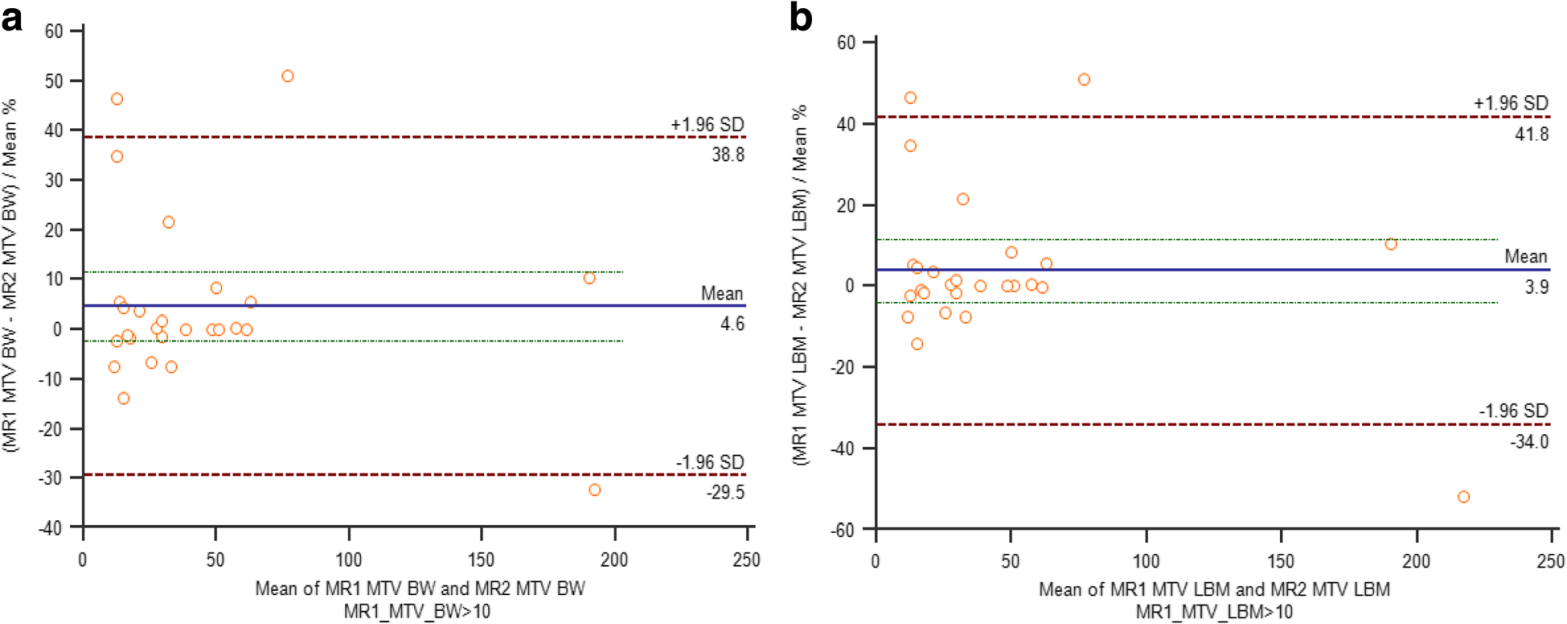
Bland-Altman plots between sequential PET/MR MTV measurements greater than 10 ml, corrected to body weight (**a**) and lean body mass (**b**)

Two sequential PET/MR SUV and SUL measurements of lesions, liver and tumor to liver ratio are shown in Table 3. Lesional SUV’s and SUL’s corrected to BW and LBM demonstrated strong positive linear correlation between two sequential PET/MR (Fig. 3). The 95% limits of agreement and mean difference expressed as percentages for lesional SUVpeak corrected to BW and LBM were below the clinically acceptable range of ±30%, but was larger for SUVmax and mean (Table 4). Representative Bland-Altman plots for SUVpeak with y-axis values expressed as percentages showed 95% limits of agreement ranging from -27.7 to 17.5 with a mean of -5.1 and -27.6 to 17.9 with a mean of -4.9 corrected to BW and LBM, respectively (Fig. 3). Lesional MTV corrected to BW and LBM demonstrated strong linear correlation between two sequential PET/MR (Fig. 4). Bland-Altman plots for MTV with y-axis values expressed as percentages showed 95% limits of agreement ranging from -42.8 to 59.1 with a mean of 8.1 and -44.3 to 59.9 with a mean of 7.8 corrected to BW and LBM, respectively (Fig. 4). After exclusion of tumors with volume less than 10ml the 95% limits of agreement ranged from -29.5 to 38.8 with a mean of 4.6 and -34 to 41.8 with a mean of 3.9 corrected to BW and LBM (Fig. 5).

**Table 3:** Two sequential PET/MR SUV and SUL measurements of tumor, liver and tumor to liver ratio

	Tumor	Liver	Ratio
SUV max			
PET/MR 1^st^	9.71 ± 5.35	2.28 ± 0.45	4.48 ± 2.74
PET/MR 2^nd^	10.33 ± 5.19	2.24 ± 0.44	4.78 ± 2.56
SUV mean			
PET/MR 1^st^	5.39 ± 2.86	1.82 ± 0.38	3.11 ± 1.82
PET/MR 2^nd^	5.92 ± 2.89	1.79 ± 0.37	3.42 ± 1.71
SUV peak			
PET/MR 1^st^	7.09 ± 3.93	2.03 ± 0.41	3.65 ± 2.21
PET/MR 2^nd^	7.42 ± 4.01	1.98 ± 0.39	3.86 ± 2.22
SUL max			
PET/MR 1^st^	7.17 ± 3.96	1.65 ± 0.31	4.55 ± 2.79
PET/MR 2^nd^	7.62 ± 3.81	1.62 ± 0.31	4.96 ± 2.93
SUL mean			
PET/MR 1^st^	3.98 ± 2.12	1.32 ± 0.27	3.16 ± 1.86
PET/MR 2^nd^	4.37 ± 2.13	1.28 ± 0.24	3.56 ± 1.89
SUL peak			
PET/MR 1^st^	5.23 ± 2.89	1.48 ± 0.28	3.69 ± 2.24
PET/MR 2^nd^	5.47 ± 2.9	1.43 ± 0.27	3.92 ± 2.23

**Table 4:** Lower and upper 95% limits of agreement and mean difference expressed as percentages for lesional SUV max, peak and mean corrected to BW and LBM

		Mean difference (%)	Limits of agreement (%)	
			Lower	Upper
PET/CT vs PET/MR				
Corrected to BW	SUVmax	7	−43.7	57.7
	SUVpeap	13.9	−27.3	55
	SUVmean	8.3	−41.1	57.6
Corrected to LBM	SUVmax	36.1	−17.2	89.3
	SUVpeak	13.7	−27.3	54.7
	SUVmean	8.4	−40.6	57.4
PET/MR vs PET/MR				
Corrected to BW	SUVmax	−7.4	−36.8	22
	SUVpeak	−5.1	−27.7	17.5
	SUVmean	−10.5	−41.8	20.7
Corrected to LBM	SUVmax	−7.2	−36.7	22.4
	SUVpeak	−4.9	−27.6	17.9
	SUVmean	−10.6	−41.5	20.4

## Discussion

Our study demonstrates strong correlation of lesional PET metrics between same day PET/CT and PET/MR and between two sequential PET/MR with good lesional SUV/L peak agreement between two sequential PET/MR.

As a new modality PET/MR test-retest repeatability and agreement with regard to SUV/L’s measurements has to be validated. Furthermore, reproducibility and agreement of PET-based variables between PET/MR and PET/CT must also be assessed as patients may swap between these modalities on follow-up studies. Principal factors that differ between PET/CT and PET/MR and might affect reliability include: different methods to create attenuation correction maps, scanning time, different PET detectors and MR hardware. Data regarding the reliability of FDG PET/MR metrics is sparse.

### Reproducibility between PET/CT with PET/MR

With regard to *lesional reproducibility*, there are conflicting results in the literature. Al-Nabhani et al. [8] have shown that lesional SUVmean measurements were approximately 10% higher on PET/MR. Pace et al. [13] have also shown that PET/MR SUVmax and SUVmean were higher in primary lesions, lymph nodes and distant metastases in the range of 34 and 21%, respectively. On the contrary, Wiesmuller et al. [6] has shown a decrease of 22% and 10% in SUVmax and SUVmean, respectively. In all studies, patients underwent PET/CT followed by PET/MR on the same day. One major assumed factor that may have influenced these results is the time interval from the radiotracer injection to scanning that was longer for PET/MR in those studies. In order to reduce the effect of injection to scan time interval we randomized the order of studies. We found good correlation of PET metrics between PET/CT and PET/MR but a wide range of limits was demonstrated on Bland-Altman plots which is considered to be clinically unaccepted.

### Repeatability between two sequential PET/MR

We found strong positive correlation for all PET metrics with clinically acceptable agreement only for lesional SUV/Lpeak. This is in accordance with Rasmussen et al. [16] who found 95% limits of agreement ranging from -12.5 to 20.4 for the different lesional SUV between two PET/MR that lies within a clinically acceptable range. A similar repeatability performance of PET/CT lesional FDG uptake was found in a meta-analysis performed by Langen et al. [17] for which 25% and 20% were found to be the limits for SUVmax and SUVmean, respectively.

**Volumetric parameters** have gained increasingly interest as prognostic factors for various cancers [18]. However, to date, only few studies have focused on MTV repeatability. We found a strong linear correlation of lesional MTV corrected to BW and LBM between PET/CT and PET/MR and between two sequential PET/MR. However, the range of 95% limits of agreement was far beyond the clinically acceptable range. Our findings are in accordance with several studies. For example, Fring et al. [19] demonstrated a repeatability range for metabolic tumor volume between two PET/CT up to 37% in non-small cell tumors greater than 4.2 ml and a range of 36% for gastrointestinal tumors [20]. Rasmussen et al. [16] found similar range between two PET/CT for head and neck squamous cell carcinoma. Interestingly, they found that the range between two PET/MR was lower than 30%. We have found similar results after exclusion of tumors with volume less than 10ml (Fig. 5).

We believe that there are two strength points in our study. First, randomization of the order of studies reduces the effect of the time interval from the radiotracer injection to scanning which has an effect on FDG uptake in lesions. Second, performing sequential PET/MR studies on the same day evaluates scanner performance with minimal effect of factors that are seen in longer interval that may influence reliability such as changes in body habitus, changes in tissues and lesional texture as a result of therapy.

Our study has several limitations. First, the number of patients is relatively small. Second, lesions determination relied on imaging findings and not on histopathology or follow up studies. Third, image analyses were performed by a single reader although with extensive experience and meticulous assessment of studies. Fourth, PET acquisition time was different between PET/CT and PET/MR which may affect SUV measurements. This, however, resembles reality where PET acquisition time in PET/MR is determined by the length of MR sequences and is longer than PET/CT.

## Conclusions

PET/MR SUV/L peak has a clinically acceptable repeatability performance and can be used to evaluate the response to treatment. PET/MR MTV measurements have a larger limit range that is inversely related to the volume of the lesion. Further studies are warranted to evaluate the reproducibility and repeatability of other imaging systems and to consolidate our findings.

## Abbreviations

BW: Body weight
CTAC: PET/CT attenuation correction
FDG: Fluorine 18 fluorodeoxyglucose
FS: Fat suppression
HASTE: Half-fourier acquisition single shot turbo spin echo
IR: Inversion recovery
LBM: Lean body mass
MRAC: PET/MR attenuation correction
MTV: Metabolic tumor volume
OSEM: Ordered subset expectation maximization
SUL: SUV for lean body mass
SUV: SUV for body weight
T/L: Tumor to liver ratio
VIBE: Volumetric interpolated breath-hold examination
VOI: Volume of interest
